# The Michigan Collaborative for Type 2 Diabetes (MCT2D): Development and implementation of a statewide collaborative quality initiative

**DOI:** 10.1186/s12913-024-11520-z

**Published:** 2024-10-17

**Authors:** Lauren Oshman, Neha Bhomia, Heidi L. Diez, Jonathan Gabison, Sherri Sheinfeld Gorin, Dina H. Griauzde, Rina Hisamatsu, Michael Heung, Cornelius D. Jamison, Katherine Khosrovaneh, Noa Kim, Joyce M. Lee, Kara Mizokami-Stout, Rodica Pop-Busui, Jacqueline Rau, Jacob Reiss, Rajiv Saran, Larrea Young, James E. Aikens, Caroline Richardson

**Affiliations:** 1 University of Michigan, Ann Arbor, MI USA; 2 Brown University, Providence, Rhode Island USA; 3 VA Ann Arbor Healthcare System, Ann Arbor, MI USA

**Keywords:** Quality Improvement, Type 2 diabetes, Primary care, Collaborative, Resources, Value-based reimbursement

## Abstract

**Background:**

Type 2 diabetes (T2D) is one of the most prevalent chronic diseases worldwide and a leading cause of cardiorenal disease and mortality. Only one-third of individuals with T2D receive care as recommended by the American Diabetes Association’s clinical practice guidelines. Effective strategies are needed to accelerate the implementation of guideline concordant T2D care.

**Methods:**

The Michigan Collaborative for Type 2 Diabetes (MCT2D) is a statewide population health collaborative quality initiative (CQI) developed to improve the care of all people with T2D in Michigan. MCT2D has developed a learning health system with physician organizations and their constituent practices to support quality improvement initiatives focused on (1) improving use of guideline-directed pharmacotherapy to improve cardiorenal outcomes, (2) increasing evidence-based use of continuous glucose monitoring, and (3) supporting use of lower carbohydrate eating patterns.

**Results:**

Between 2021 and 2022, MCT2D recruited 28 of the 40 Michigan-based physician organizations participating in Blue Cross’ Physician Group Incentive Program with 336 constituent practices and 1357 physicians in primary care (304), endocrinology (21) and nephrology (11). In January 2022, baseline data included a sample of 96,140 unique individuals with T2D. The baseline HbA1c was ≤ 7.0% for 66.3% of patients (*n* = 32,787), while 14.9% of patients had a most recent HbA1c ≥ 8.0% (*n* = 7,393). The most recent body mass index (BMI) was ≥ 30.0 for 64.8% of patients (*n* = 38,516).

**Discussion:**

MCT2D has organized a statewide collaborative to recruit and engage a diverse and large set of physician organizations and their constituent practices. This is a promising opportunity to accelerate adoption of guideline-concordant care for people with T2D and may be a model for other state or regional collaboratives. Future directions include specific evidence-based interventions targeted at reducing diabetes-linked comorbidities and associated healthcare costs as well as strategies focused on T2D prevention among at-risk populations.

**Supplementary Information:**

The online version contains supplementary material available at 10.1186/s12913-024-11520-z.

## Introduction

Type 2 diabetes (T2D) is a common, costly, and disabling chronic disease that affects 11% of adults in the United States [1]. Its most common complications include atherosclerotic cardiovascular disease (ASCVD), heart failure, chronic kidney disease, diabetic neuropathy and diabetic retinopathy [2]. Obesity is increasingly recognized as a key risk factor for T2D [3]. With 38% of Americans affected by prediabetes and 42% affected by obesity, the prevalence of T2D is projected to increase to 17.9% among US adults by 2060 [4–6]. Multiple clinical practice guidelines for T2D now encourage the use of multiple strategies to support improved cardiorenal outcomes, glycemic control, and weight loss [7, 8]. Such strategies include use of glucagon-like peptide-1 receptor agonists (GLP-1 RAs) and sodium-glucose cotransporter-2 inhibitors (SGLT2is) [9–12], continuous glucose monitors (CGMs) combined with pharmacological interventions and/or lifestyle modifications [13, 14], and lower carbohydrate eating patterns (defined as < 130 total grams of carbohydrate per day) [15]. 

Unfortunately, only 34% of individuals with T2D receive guideline-concordant care as recommended by the American Diabetes Association Standards of Care [16, 17]. Treatment focused on complication reduction and weight loss has been particularly slow to diffuse into primary care settings, where over 90% of T2D care occurs [18]. Finally, quality improvement efforts have been significantly hindered by the COVID-19 pandemic, which profoundly disrupted ambulatory care revenue, delivery, and staffing [19]. 

Prior T2D quality improvement has largely focused on increasing the frequency of laboratory testing (e.g., HbA1c, urine albumin), glycemic control, statin use, and blood pressure control among people with T2D [20]. Such initiatives have also focused on improving glycemic control but without explicit use of strategies to reduce cardiorenal complications and/or support weight loss through use of newer medications (i.e., GLP-1 RAs, SGLT2is), CGMs, and/or lower carbohydrate eating patterns. To address this gap, Blue Cross and Blue Shield of Michigan (BCBSM) sponsored the development of a T2D collaborative quality initiative in 2021.

**Fig. 1:**
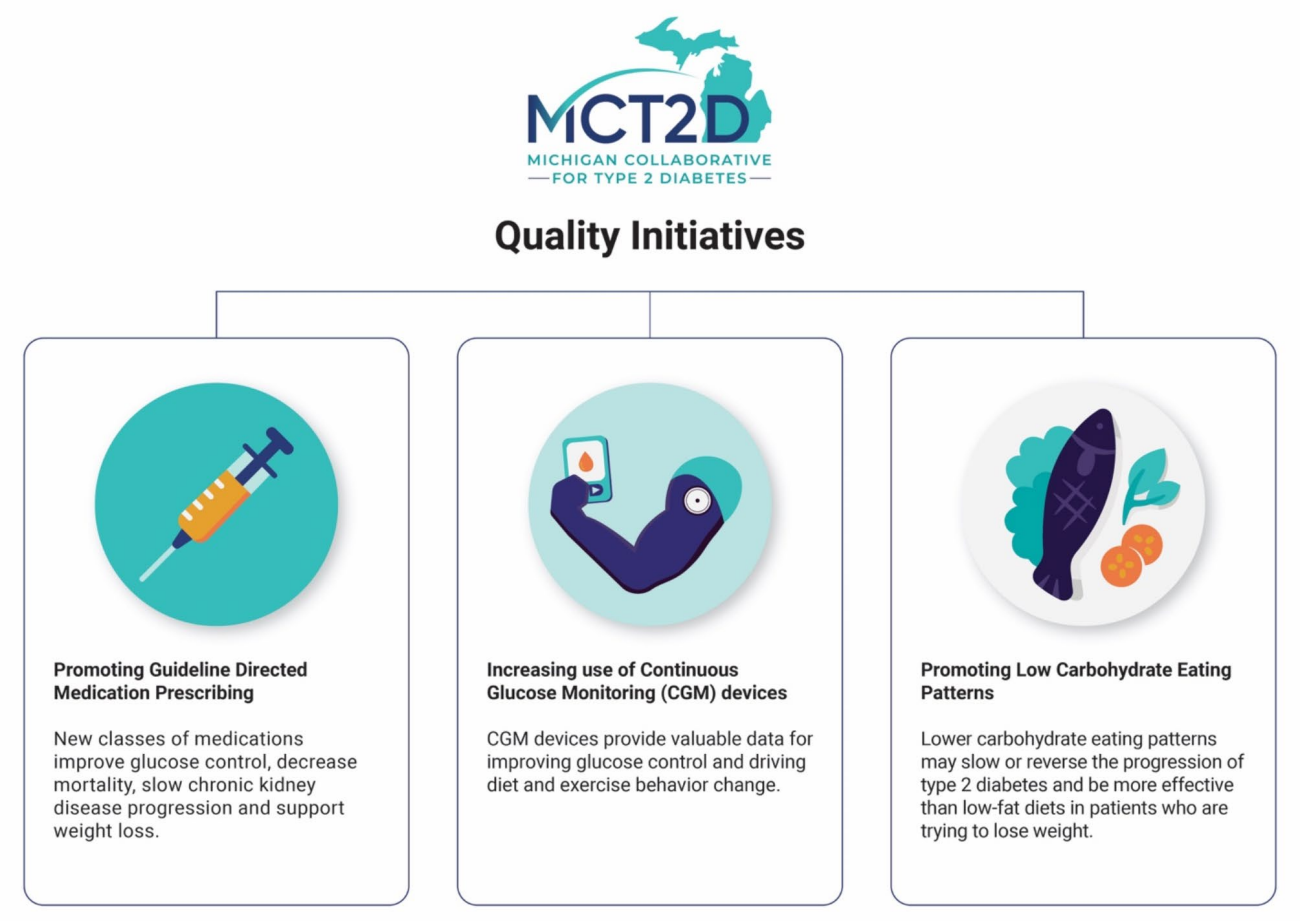
MCT2D quality improvement initiatives

The goal of the Michigan Collaborative for Type 2 Diabetes (MCT2D) is to prevent the complications of type 2 diabetes by fostering a collaborative community of primary care, endocrinology, and nephrology clinicians and patients to accelerate the equitable implementation of evidence-based diabetes care for all patients in Michigan. Our ultimate mission is preventing and remitting T2D and its complications in the state of Michigan. To accelerate the dissemination and implementation of new evidence-based practices for T2D into primary care settings, MCT2D selected three initial evidence-based quality improvement (QI) initiatives that are often underutilized yet highly effective for improving outcomes for patients with T2D: (1) guideline-directed use of pharmacotherapy to improve cardiorenal outcomes, including appropriate prescribing of SGLT2i and GLP-1 RA medications, (2) evidence-based use of continuous glucose monitoring (CGM), and (3) lower carbohydrate eating patterns [Fig. 1]. Here we describe the origin, structure, and function of MCT2D.

## Methods

### History of collaborative quality initiatives (CQIs)

Blue Cross Blue Shield of Michigan (BCBSM) and its HMO (Blue Care Network) began supporting “Collaborative Quality Initiatives” (CQIs) in 1997 as a means of addressing specific health care quality concerns. At present, there are 22 Blue Cross CQIs and most focus on acute, hospital-based care including surgical complication reduction [21, 22]. Recognizing the unmet need to further improve care for high-cost ambulatory chronic conditions, BCBSM established MCT2D in 2021.

### Quality improvement initiatives

#### Drug classes with cardio-renal benefit

GLP-1 RA’s and SGLT2i’s are two classes of medication that have emerged over the last two decades which improve glycemic control and provide primary or secondary prevention of serious cardiovascular and renal complications [23, 24]. Starting in 2016, select medications from the SGLT2i class were FDA-approved for risk reduction of major adverse cardiovascular events (MACE) in patients with T2D and cardiovascular disease or with multiple cardiovascular disease risk factors [24, 25]. Several SGLT2 and SGLT1,2 inhibitors are now also approved to prevent the progression of chronic kidney disease and improve heart failure and cardiovascular death outcomes in T2D [9–11, 26, 27]. 

The American Diabetes Association’s (ADA) 2023 Standards of Care recommends that patients with T2D and established ASCVD or two or more ASCVD risk factors receive either a SGLT2i or GLP-1 RA with proven cardiovascular benefit as the first line medication for glycemic control. This move away from metformin as the first-line medication for every individual with T2D represents a major shift in guideline directed-care [28]. The ADA Consensus for Heart Failure in Diabetes endorsed by the American College of Cardiology (ACC) recommends that for people with T2D and heart failure, even if asymptomatic (Stage B), SGLT2i are an expected element of care [10, 24, 29]. The Standards of Care recommend a GLP-1 RA for individuals with T2D and obesity rather than obesogenic medications such as insulin and sulfonylureas, which may need to be de-prescribed [9, 30]. 

Clinical uptake of drug classes with cardiorenal benefit has lagged due to clinician and insurance barriers including limited knowledge, limited visit time to discuss treatment changes, complicated prior authorization processes, drug shortages, and high drug costs that may jeopardize the financial performance of value-based insurance arrangements [31–33]. Patient barriers include reluctance toward injectable agents, side effects, lack of awareness of cardiorenal benefits, and high out-of-pocket costs [34–37]. 

#### Continuous glucose monitoring (CGM)

Clinical practice guidelines support the use of CGM in people who use insulin, based on evidence from several recent clinical trials demonstrating improvement in glycemic control, reduction in HbA1c and time in range, reduction in serious hypoglycemia episodes, and reduction in emergency care utilization [38–40]. Clinical trials also support CGM in people with T2D who do not use insulin when paired with diabetes education to improve HbA1c and time in range [14]. 

Several barriers exist to uptake and ongoing use of CGM, particularly in primary care settings. These include insufficient clinician knowledge and training on prescribing and interpreting CGM and inadequate clinic resources (including electronic health record technology) to upload CGM data. Despite clinical trial results supporting effectiveness, insurance coverage for CGM remains limited for people with T2D who are not prescribed insulin [14]. Other barriers include complicated prior authorization protocols; and lack of access to, and training of, dieticians or other clinicians in medical nutrition therapy counseling to support patients in using CGM data to change their eating patterns [41]. 

#### Lower carbohydrate eating patterns

Low- and very low-carbohydrate eating patterns are commonly defined as 50–130 g of carbohydrates per day and less than 50 g of carbohydrates per day, respectively. Lower carbohydrate eating patterns can support weight loss, glycemic control, and favorable changes in cholesterol, blood pressure, and self-reported measures of energy, hunger, and food cravings while reducing the need for medications to control chronic conditions including T2D and hypertension [42–45]. The ADA Consensus Statement for nutrition therapy for adults with T2D and prediabetes states “reducing overall carbohydrate intake for individuals with diabetes has demonstrated the most evidence for improving glycemia and may be applied in a variety of eating patterns that meet individual needs and preferences.” [15, 46] However, patients have few strategies to support their use of lower carbohydrate eating patterns if they prefer these approaches and/or have not achieved glycemic targets with higher carbohydrate meal plans [47]. 

Barriers to the use of low- and very low-carbohydrate eating approaches in primary care and endocrinology settings include clinician lack of knowledge and concerns about potential LDL cholesterol elevations [48]. Most practices lack specific resources to safely help patients initiate and sustain a carbohydrate-restricted eating pattern, including frequent nutrition counseling appointments, and timely de-escalation of anti-hyperglycemic and anti-hypertensive medications [49]. 

### CQI structure and organization

The MCT2D coordinating center is located at the University of Michigan and is supported by an infrastructure that serves several CQIs. The coordinating center team includes program directors (a family physician researcher (LO) and an academic clinical pharmacist (HD)), program managers, information technologists, data analysts, human-centered designers, graphic designers, and communications professionals. MCT2D partners with nephrology program directors (RS, MH, JWN), an endocrinology initiative director (RPB), and content experts with expertise in T2D, obesity, quality improvement, implementation science, and health equity. The MCT2D steering committee is composed of physician organization and practice champion representatives and provides feedback and guidance on performance metrics, agendas for participant meetings, and the overall direction of the collaborative.

MCT2D enrolls physician organizations to participate in the collaborative, who then enroll participating physician practices. A physician organization is an entity formed by physicians to pursue common interests in a collaborative manner. Participation in MCT2D requires a minimum number of BCBSM patients with type 2 diabetes at a participating practice. The physician organization facilitates the success of their participating practices by leading quality improvement efforts, disseminating information and education, and supporting data exchange. MCT2D recruits new cohorts of physician organizations on an annual basis. The program enrolled the first cohort of participants in September 2021, the second in 2022, and a third cohort will be recruited in 2024 with planned recruitment every other year.

Participation in MCT2D requires a minimum number of BCBSM patients with type 2 diabetes at a participating practice. Each participating practice must designate a practice champion. This role can be fulfilled by a physician, physician assistant, nurse practitioner, nurse, clinical pharmacist, diabetes educator, or care manager. Practice champions attend MCT2D educational sessions about improving care for patients with T2D, disseminate educational content to their practice team, work with leadership to implement practice change, and champion improvement efforts at their practice.

MCT2D facilitates community and engagement among participating practices by hosting regional in-person meetings for practice clinical champions twice per year. Practices are divided into seven geographical regions across Michigan to reduce travel and time burden for participants and facilitate local and regional relationships. At regional meetings, participants share best practices, provide group learning opportunities, and identify new topics for the collaborative to address. Physician organizations and practices contribute to the meeting agenda to help ensure their needs are being met, and the meeting combines both presentations and participant discussion.

In addition to recruiting primary care practices in year one, MCT2D also encouraged POs to recruit a subset of endocrinology and nephrology specialty sites that frequently share patients with participating primary care practices. In year two, MCT2D broadened its recruitment efforts to include all eligible endocrinology and nephrology practices in the PO. Participating nephrologists are asked to focus on prescribing SGLT2is and GLP-1 RAs and to regularly test urine albumin, while participating endocrinologists are asked to focus on all three quality improvement initiatives, as well as coordinating care with primary care practices and other specialists.

### Patient advisory board

MCT2D aims to place the patient’s voice at the center of T2D through a patient advisory board. Patient advisors are recruited by MCT2D participating practices and meet every other month over video teleconferencing and provide feedback about quality improvement initiatives, educational materials, and the strategic direction of the collaborative. Additionally, they contribute stories about their experiences with T2D that are shared on social media. Patient advisors are invited to present at regional and collaborative-wide meetings. They are compensated $25 for each meeting they attend and are compensated for travel for meeting attendance.

### MCT2D participation requirements and participant incentives

#### Physician organizations

Requirements for participation in MCT2D include attendance at monthly meetings with the coordinating center to track progress; completing progress reports; engaging their participating practices and guiding their quality improvement efforts; and sharing feedback with the coordinating center (see Appendix 1). To support active engagement in meeting MCT2D requirements, each physician organization receives financial rewards and incentives from BCBSM to support the necessary infrastructure for implementation and administration of MCT2D’s initiatives.

#### Practices and physicians

Practice requirements center around empowering the practice clinical champions to disseminate information and partnering with their physician organization to implement and evaluate change within their practice. Examples of practice-level requirements include completion of baseline practice change readiness questionnaires and needs assessments; participation in regional meetings; sharing best practices; participating in activities of a learning community; and completing yearly progress reports. In addition, each practice clinical champion is expected to participate in six initial hours of education about MCT2D’s initiatives and to disseminate this education to team members at their practices. Physician requirements center around engagement activities, including participation in education and implementation and evaluation of the MCT2D initiatives.

As a reward for their efforts, BCBSM has implemented a value-based reimbursement (VBR) incentive for participating physicians that meet MCT2D engagement requirements. Primary care physicians who meet the current requirements are eligible to receive a VBR incentive through an annual fee schedule increase on specific professional claims for their BCBSM commercial members. Specialist physicians who met all the requirements received a similar VBR incentive for their BCBSM commercial members. MCT2D has engaged other payers which are working to create incentives for their participating physicians.

### MCT2D as a learning health system

The Agency for Healthcare Research and Quality (AHRQ) defines a learning health system as a system “in which internal data and experience are systematically integrated with external evidence, with that knowledge put into practice.” [50] MCT2D functions as a learning health system by collecting physician organization, practice, and individual data, analyzing this data, and engaging with the physician organization and their practice teams to improve care through practice change [51]. 

MCT2D developed a learning community to enhance participant engagement and implementation of evidence-based care and incentivized participants to share barriers to and opportunities for improving T2D care through surveys and discussion groups. The coordinating center then created tailored tools and resources that were created by content experts and then refined through clinician and patient feedback and made available at www.mct2d.org (see Appendix 2). Examples of patient tools include patient medication education tip sheets, a patient low carb diet starter guide, and a patient CGM-based lifestyle change guide. Examples of clinical tools include dose titration and precautions for GLP-1 RA and SGLT2i prescribing; a comprehensive guide to insurance coverage for medications and CGMs; and templates for prior authorization and clinical documentation. A component of BCBSM’s VBR physician incentive is related to participating physicians sharing best practices and successes through MCT2D newsletters, webinars, and learning community blog posts.

MCT2D provides education through biannual regional meetings, annual collaborative-wide meetings, and monthly lunchtime learning webinars. Topics for webinars are generated from periodic needs assessments of participants. Practice champions and physicians in the practice that meet their participation obligations are incentivized both through BCBSM’s VBR incentive program described above as well as the ability to earn continuing education credit.

### User centered design

MCT2D emphasizes user centered design, including users’ input in product design and development, as a key tactic for quality improvement. We draw from a highly diverse participant base that includes rural and urban practices, large and small physician organizations, healthcare professionals with varying specialties and backgrounds, and patients of different socio-demographics from across the state. To ensure that MCT2D tools and resources fit the needs of this diverse group, the collaborative uses participatory design methods to actively involve stakeholders in the design and development process. These methods include: 30-minute one-on-one user experience feedback interviews regarding the design of the MCT2D patient data dashboard and online resource library; feedback surveys of patient and provider tools; 15-minute one-on-one and small group lightning discussions, process mapping workshops; and open-ended brainstorming sessions. Both qualitative and quantitative data are collected using these methods; the data are reviewed on an ongoing basis. This information is incorporated into all design decisions including tool updates, user interface changes, feature and tool additions, and future planning.

### Data sources and population health registry

BCBSM initiated a collaboration between data registry and reporting platform design and implementation experts, Michigan’s primary Health Information Exchange, the Michigan Health information Network, and MCT2D to create a population health registry to measure and improve population health. Participating physician organizations are encouraged to utilize an electronic health record that provides data interoperability with the Michigan Health information Network. The registry includes claims data from Blue Cross Blue Shield of Michigan and Blue Care Network and clinical data from Michigan’s Physician-Payer Quality Collaborative, an initiative that facilitates sharing of all-payor all-patient core quality measure data. Currently MCT2D only receives BCBSM and BCN Physician-Payer Quality Collaborative data, but work is in progress to incorporate all payor data. Examples of available data fields include laboratory values such as HbA1c, estimated glomerular filtration rate, urine albumin creatinine ratio; medication claims for antihyperglycemic agents, statins, angiotensin converting enzyme inhibitors and angiotensin receptor blockers, and socio-demographic characteristics such as patient age and sex. To address limitations in access to all payor claims data, future iterative expansion of Physician-Payer Quality Collaborative data will include a record of prescribing for medications and CGM [52]. 

Currently, MCT2D’s collaborative wide database includes individuals over 18 years old who meet at least one of the current definitions for T2D: (1) ICD-9/ICD-10 diagnostic code for T2D, (2) HbA1C of 6.5% or greater, (3) prescribed an anti-hyperglycemic medication. Individuals with an ICD-9/ICD-10 diagnostic code for T1D are excluded.

Dashboards are available with patient, practice, physician organization, and collaborative-level data. Attribution models match patients with their primary care and specialty physicians. The patient data dashboard allows practices to identify patients with treatment gaps and opportunities for improvement in T2D care and target these patients for individualized treatment. The collaborative-wide, practice and physician organization level reporting views allow the tracking of population level diabetes metrics and comparisons. These offer opportunities for collaborative-wide improvement through sharing best practices from high performers. Reports are also used at regional and collaborative wide meetings to enable physician organization leaders and practice clinical champions to identify barriers and map solutions regarding MCT2D’s initiatives.

### Health equity

Recognizing that substantial health disparities occur in both the development and treatment of T2D, MCT2D is working to ensure that implementation of the three quality initiatives does not widen existing gaps in access to care, insurance barriers, and race/ethnic disparities [53, 54]. To ensure equitable representation of priority communities, MCT2D sought to enroll federally qualified health centers and safety net clinics in our recruitment efforts and will include all-payer data (including Medicaid) to engage participating practices in health equity-focused improvement efforts. As an example of our current health equity projects, MCT2D collaborated with another CQI program, Healthy Behavior Optimization for Michigan, on a pilot of food delivery and diabetes nutrition education for patients with food insecurity through our practice network [55]. 

### Pre-participation change readiness assessment and progress reporting

We assessed physician organization readiness to participate in MCT2D with a semi-structured qualitative interview guide using the Consolidated Framework for Implementation Research (CFIR) domains (see Appendix 3) [56]. The coordinating staff conducted interviews with leaders from each physician organization, covering characteristics of the inner and outer settings, intervention characteristics, processes, and characteristics of the involved individuals. Two team members performed rapid content analysis to identify key barriers and facilitators to guide the development of our collaborative learning community [57]. We assessed practice readiness to participate in MCT2D with a 32-question quantitative survey for practice champions based on CFIR domains. Questions also included familiarity with MCT2D goals, level of communication and support from the physician organization, and confidence related to implementation of MCT2D’s initiatives (see Appendix 4). Survey response items used a 5-point Likert scale (Very much to Not at all); responses were collapsed into two categories (Very much and Mostly vs. Moderately, Somewhat, Not at all) for statistical analysis. Descriptive statistical analyses were conducted using SAS 9.4 [58]. 

## Results

**Fig. 2:**
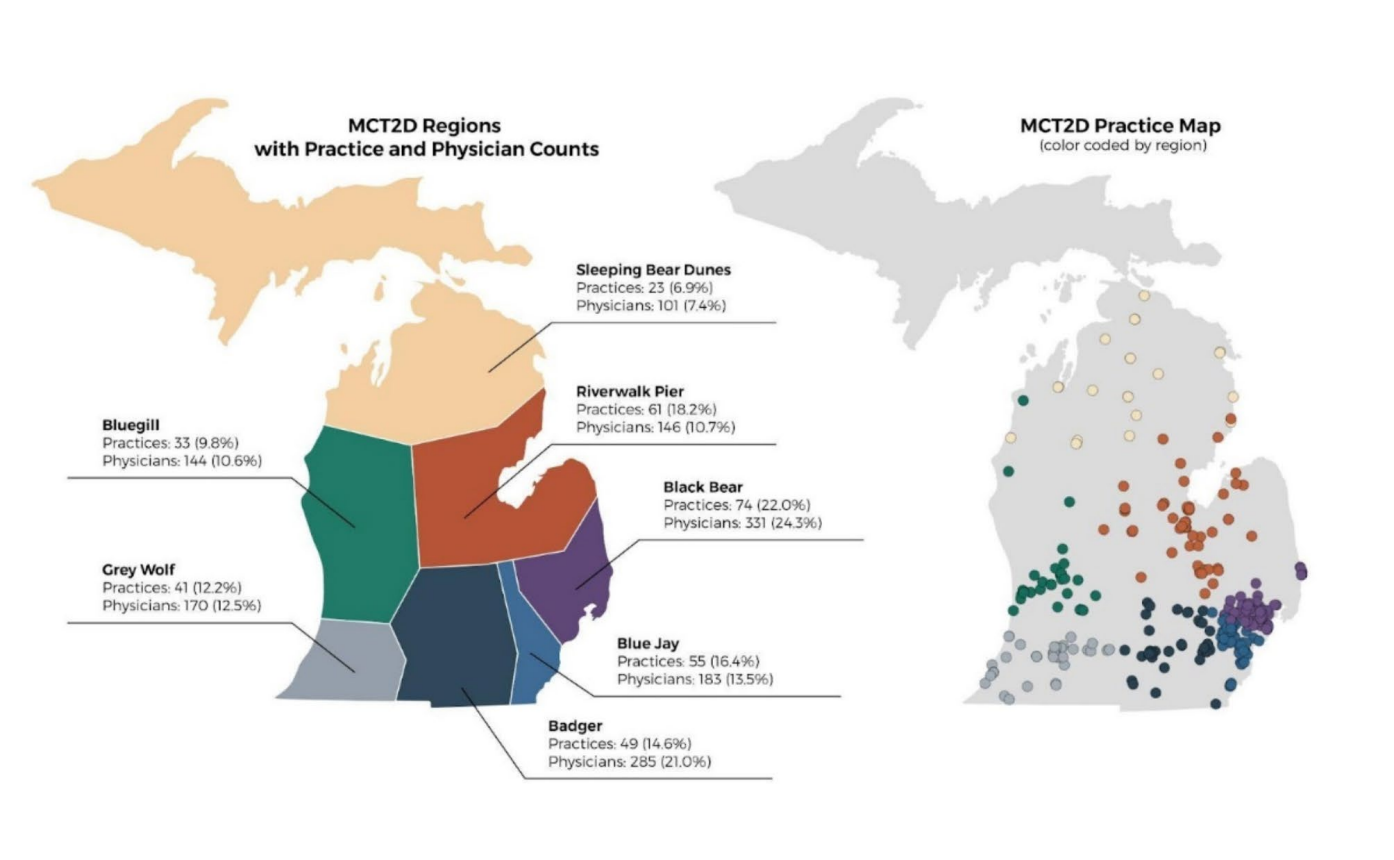
MCT2D regional practice map

MCT2D currently consists of 28 physician organizations representing a total of 336 practices and 1357 physicians. Participating organization and practice baseline characteristics are shown in Table 1. Among the practices, 6.3% are Endocrinology practices (*N* = 21), 3.3% Nephrology (*N* = 11), and 90.5% primary care (*N* = 304), and 36% of primary care practices are independently owned. EPIC is the electronic medical record (EMR) most used, encompassing 43% of the practices. Practices were divided into 7 major regions (see Fig. 2 for the Regional Practice Map), based on their geographic location; each is named by the participating practice champions. The regional divisions facilitate in-person meetings and cross-collaborative engagement.

**Table 1:** Characteristics of MCT2D practices

Characteristics	*N* (%)
Physician Organization (PO) type Employed Independent	28 18 (64.3%) 10 (35.7%)
Practice Specialty Type PCP Endocrinology Nephrology	336 304 (90.5%) 21 (6.3%) 11 (3.3%)
Physician Count by Speciality Primary Care Endocrinology Nephrology	1357 1185 (87.3%) 82 (6.0%) 90 (6.6%)
Practice Primary Payor^a^ (*N* = 282)	
Blue Cross Various Medicare Commercial Medicaid Priority Health	95 (33.7%) 82 (29.1%) 62 (22.0%) 30 (10.6%) 10 (3.5%) 3 (1.1%)
Practice Electronic Medical Record (*N* = 281)	
EPIC eClinicalWorks Other^b^ Allscripts Athena Aprima Cerner Greenway NextGen	122 (43.4%) 36 (12.8%) 32 (11.4%) 31 (11.0%) 28 (10.0%) 10 (3.6%) 10 (3.6%) 6 (2.1%) 6 (2.1%)

^a^Clinical champions were asked to respond to an open-ended question “*Who is your primary payor?”* Some provided more than one insurance plan; therefore, they were categorized as various

^b^EMRs used by less than 2% of the MCT2D practices categorized as “other” include: Advanced MD, Amazing Charts, Dr. Chrono, e Thomas, E-Medical Notes, Genius Solutions, Health Fusion, Intelligent Medical Systems (IMS), Ipatientcare, MEDINFORMATIX, ModuleMD, ParkNet, Practice Fusion, Practice Partner, R & G Web Chart, Sevocity, Triarq, Veradigm, Waiting Room Solutions, Other

Pre-Participation Change Readiness Assessment results are shown in Table 2 for 262 practice champions and Table 3 for 35 physician organizations. Most practice champions reported that they very much or mostly understood MCT2D’s goals and objectives (175, 66.8%). Practice champions reported the highest level of confidence implementing the guideline-directed medication and lower carbohydrate eating patterns initiatives (169, 64.5%) compared to the CGM initiative (153, 58.4%). Ratings were uniformly high for perceived commitment toward the success of MCT2D (236, 90.1%) and support for MCT2D’s goals (222, 84.7%) from the physician organization. Among the 35 qualitative responses from physician organizations, lack of resources for data sharing (*n* = 7) and clinician engagement (*n* = 7) were the top concerns regarding practice change readiness.

**Table 2:** Practice champion pre-participation change readiness survey results (*N* = 262^a^)

	*N* (%) or Mean (+/- SD)^b^
I understand MCT2D’s goals and objectives.	175 (66.8%)
I am confident regarding MCT2D’s QI initiatives:	
Cardiorenal benefit medication classes	169 (64.5%)
CGM	153 (58.4%)
Lower carbohydrate eating patterns	169 (64.5%)
PO is committed to success of MCT2D	236 (90.1%)
PO supports us to fulfill MCT2D goals.	222 (84.7%)

^a^196 out of 252 (77.7%) of Cohort 1 practices and all 66 Cohort 2 practices consented to use of this data in publication. One Physician Organization submitted one single response to represent all practices

^b^Practice champions answering very much or mostly to a Likert scale (very much, mostly, moderately, somewhat, not at all)

**Table 3:** Physician organization pre-participation change readiness survey results: biggest anticipated challenge related to MCT2D participation (*n* = 35)^a^

Theme	Exemplar Quote	Frequency
**Lack of Resources**	*Probably the data sharing aspect of it. We have very limited resources when it comes to putting files together for data and sending the information.*	**7**
**Clinician Engagement and Change Fatigue**	*Also*,* our PO does have lots of physicians who have been in practice for lots of years*,* and while the collaborative is exciting*,* sometimes those physicians that have been around for years kind of take the wait and see attitude. Sometimes the primary care physician in trying to do the best thing for the individual patient in front of them and is not necessarily seeking the next new thing*,* so the technology of glucose monitoring may not be something that our physicians embrace as exciting as it may be.*	**7**
**Patient Activation**,** Motivation**,** and Engagement**	*Engagement of patients to the program since getting patients engaged can be difficult when you’re talking about lifestyle changes*,* especially changing habits*,* that’s not always easy (not specific to this initiative*,* just in general).*	**6**
**Cost of GLP1-RAs/SGLT2is and CGM Insurance Coverage Limitations**	*One thing we’re really thinking with effective disease management when it comes to pharmacy*,* the medications are costly*,* every major barrier.*	**6**
**Busy Physician Schedules**	*The challenge for the practice is that our practices are very busy and have a national staffing shortage due to the pandemic.*	**4**

^a^Cohort 1 physician organizations only

Collaborative wide baseline data results are shown in Table﻿ 4. The current total patient sample represented in the MCT2D data dashboard is 96,140 unique patients who meet the identified data definition criteria for type 2 diabetes. Among these, 50.4% were female and 48.3% were at least 65 years of age. Pre-implementation baseline data from January 2022 indicate that 51.5% of the total patient sample had a HbA1c value transmitted via the Michigan Health information Network to MDC. The baseline HbA1c was ≤ 7.0% for 66.3% of patients (*n* = 32,787), while 14.9% of patients had a most recent HbA1c ≥ 8.0% (*n* = 7,393). The most recent body mass index (BMI) was ≥ 30.0 for 64.8% of patients (*n* = 38,516).

**Table 4:** Collaborative wide population health registry baseline data^a^

Total Patient Sample (*n* = 96,140)	*N* (%)
*Demographic*	Total
Gender	
Female Male Unknown	48,424 (50.4%) 47,713 (49.6%) 3 (0.0%)
Age (years)	
≥ 65 < 65	46,415 (48.3%) 49,725 (51.7%)
*HbA1c* ^*b*^	*N* = 49,472
< 7% 7-7.9% ≥ 8.0%	32,787 (66.3%) 9,292 (18.8%) 7,393 (14.9%)
*BMI* ^*c*^	*N* = 59,445
≤ 24.99 25.0-29.99 ≥ 30.0	5,748 (9.7%) 15,181 (25.5%) 38,516 (64.8%)
*Medications for glycemia and CGM* ^*d, e*^	
Carve out or unknown	24,536 (25.5%)
No carve out^d^	71,604 (74.5%)
Medication utilization^e^	
GLP-1 receptor agonist	8,187 (11.4%)
SGLT2i inhibitor	5,828 (8.1%)
Insulin basal bolus (Short + Intermediate or Long-Acting Insulin) Basal only (Intermediate or Long-Acting Insulin)	6,776 (9.5%) 6,575 (9.2%)
Metformin	29,826 (41.7%)
Sulfonylurea	9,082 (12.7%)
CGM^f^	1,542 (2.2%)

^a^Data was extracted from January 1, 2018 - January 1, 2022

^b^Denominator of the percentages is the total participant sample who have HbA1c values (*N* = 49,472). Data Manipulation: HbA1c values less than 4.0% and greater than 20.0% were excluded from analyses

^c^Denominator of the percentages is the total participant sample who have BMI values (*N* = 59,445). BMI values less than 15 and greater than 150 were excluded from analyses

^d^Some groups/employers purchase separate pharmacy coverage through different vendors. This is called a “pharmacy carve out.” These vendors do not send claims data to MCT2D. Therefore, MCT2D is only able to provide pharmacy claims data on participants with BCBSM pharmacy coverage

^e^Medications are displayed only for participants with no pharmacy carve out, total *n* = 71,604

^f^Continuous glucose monitor (CGM) data is sourced from medical and pharmacy claims

Pharmacy claims are available for 74.5% of patients (*n* = 71,604) and unavailable for 24,536 patients due to lack of data from insurance plans with pharmacy carve-outs. Of the patients with pharmacy claim data available at baseline in January 2022, 11.4% (*n* = 8,187) of patients had at least one pharmacy claim for a GLP-1 RA medication and 8.1% (*N* = 5,828) had at least one pharmacy claim for an SGLT2i. Among the patients with pharmacy or medical claims, 1,542 (2.2%) had at least one claim for a CGM.

## Discussion

The Michigan Collaborative for Type 2 Diabetes is a statewide collaborative quality initiative focused on improving treatment and outcomes for people with T2D by engaging primary care, endocrinology, and nephrology practices in delivering guideline-concordant care. In the first three years, we established the collaborative structure, enrolled a robust membership of diverse physician organizations and practices in primary and specialty care, and built a statewide data registry scaffold to support data reporting for quality improvement.

Pre-participation change readiness results indicate a high level of practice and organization interest and engagement in the MCT2D program. Factors contributing to these results may be MCT2D’s user-centered design approach to the creation of a learning community, patient and provider focused tools and education, and the input of our patient advisory board [59]. In addition, BCBSM’s VBR incentive model, which rewards MCT2D participants, may be important in driving quality improvement. While Blue Cross is the sole funder of the MCT2D program, the CQI program was designed to provide data, tools and resources to be used by health care professionals for all patients, regardless of payer, to meet the needs of the entire population with T2D.

Limitations of MCT2D include the current lack of Medicaid and other health insurer and data, key elements including diagnoses codes, and claims data from patients with pharmacy carve-outs from our current data registry. With future updates, the population health registry will include data from multiple health insurers and provide robust reporting that can inform quality improvement and track performance for value-based reimbursement payments. Future anticipated data elements include patient race and ethnicity, zip code, social determinants of health indicators, and insurance and payer information. With these data, MCT2D will be able to focus on health equity as a key component of our initiatives. The addition of diagnoses codes for heart failure, other types of cardiovascular diseases, kidney disease and data elements for key laboratory variables such as serum creatinine and urine albumin will assist us in targeting quality improvement focused on patients at the highest risk for cardiorenal complications.

### Future directions

Based on strategic planning feedback from collaborative members, MCT2D plans to expand work to address prediabetes, obesity, and patients with newly diagnosed T2D through new quality improvement initiatives and partnerships with other CQI programs, such as the Michigan Bariatric Surgery Collaborative, in our effort to prevent and remit T2D. To further address barriers identified by our patient advisory board and practice champions, the MCT2D coordinating center and its members are building an advocacy coalition to address treatment costs, coverage for evidence-based treatments, and expanded financial resources through the sharing of patient and clinician stories in partnership with the American Diabetes Association. To improve health equity, MCT2D will continue to partner with safety net and federally qualified health center clinics, to expand our data sources, and to advocate for changes in insurance coverage to cover evidence-based interventions to prevent and remit T2D and reduce diabetes complications.

## Conclusion

MCT2D is a statewide collaborative quality initiative that has recruited and engaged a large and diverse group of physician organizations and their affiliated primary care and specialty practices to address quality improvement opportunities for people with type 2 diabetes across Michigan. MCT2D’s structure and methods may inform future statewide or regional quality initiatives to improve treatment and outcomes for patients with T2D.

## Electronic supplementary material

Below is the link to the electronic supplementary material.

Supplementary Material 1: Appendix 1

Supplementary Material 2: Appendix 2

Supplementary Material 3: Appendix 3

Supplementary Material 4: Appendix 4

## Data Availability

Data supporting practice information and physician organization information for Tables 1 through 3 are not available as they are either proprietary to participation in a BCBSM value-based care program or may identify individual practices and physician organizations. Raw patient data that supports Table 4 is not available based on the data sharing agreement of each physician organization sharing data with MCT2D.

## Abbreviations

ADA: American Diabetes Association
AHRQ: Agency for Healthcare Research and Quality
ASCVD: Atherosclerotic Cardiovascular Disease
BCBSM: Blue Cross Blue Shield of Michigan
BCN: Blue Care Network
BMI: Body Mass Index
CFIR: Consolidated Framework for Implementation Research
CGM: Continuous Glucose Monitor
COVID-19: Coronavirus Disease
CQI: Collaborative Quality Initiative
FDA: Food and Drug Administration
GLP-1RA: Glucagon-Like Peptide-1 Receptor Agonists
HbA1c: Hemoglobin A1C
IRB: Institutional Review Board
MACE: Major Adverse Cardiovascular Events
MCT2D: Michigan Collaborative for Type 2 Diabetes
SGLT2i: Sodium-Glucose Cotransporter-2 Inhibitors
T1D: Type 1 Diabetes
T2D: Type 2 Diabetes
VBR: Value Based Reimbursement

## References

[1] Centers for Disease Control. Prevalence of Both Diagnosed and Undiagnosed Diabetes. 2022. https://www.cdc.gov/diabetes/data/statistics-report/index.html

[2] Raghavan S, Vassy JL, Ho Y, et al. Diabetes Mellitus–related all-cause and Cardiovascular Mortality in a national cohort of adults. J Am Heart Assoc. 2019;8(4):e011295. 10.1161/JAHA.118.011295. 30776949 10.1161/JAHA.118.011295 PMC6405678

[3] Nianogo RA, Arah OA. Forecasting obesity and type 2 diabetes incidence and Burden: the ViLA-Obesity Simulation Model. Front Public Health. 2022;10:818816. 10.3389/fpubh.2022.818816. 35450123 10.3389/fpubh.2022.818816 PMC9016163

[4] Prevalence of Prediabetes Among Adults | Diabetes | CDC, September. 21, 2022. Accessed July 13, 2023. https://www.cdc.gov/diabetes/data/statistics-report/prevalence-of-prediabetes.html

[5] Obesity is a Common, Serious, and Costly Disease. Centers for Disease Control and Prevention. July 20, 2022. Accessed July 13. 2023. https://www.cdc.gov/obesity/data/adult.html

[6] Lin J, Thompson TJ, Cheng YJ, et al. Projection of the future diabetes burden in the United States through 2060. Popul Health Metr. 2018;16(1):9. 10.1186/s12963-018-0166-4. 29903012 10.1186/s12963-018-0166-4 PMC6003101

[7] El Sayed NA, Aleppo G, Aroda VR, et al. Pharmacologic approaches to Glycemic Treatment: standards of Care in Diabetes—2023. Diabetes Care. 2022;46(Supplement1):S140–57. 10.2337/dc23-S009. 10.2337/dc23-S009 PMC9810476 36507650

[8] Samson SL, Vellanki P, Blonde L, et al. American Association of Clinical Endocrinology Consensus Statement: Comprehensive Type 2 Diabetes Management Algorithm – 2023 update. Endocr Pract. 2023;29(5):305–40. 10.1016/j.eprac.2023.02.001. 37150579 10.1016/j.eprac.2023.02.001

[9] Palmer SC, Tendal B, Mustafa RA, et al. Sodium-glucose cotransporter protein-2 (SGLT-2) inhibitors and glucagon-like peptide-1 (GLP-1) receptor agonists for type 2 diabetes: systematic review and network meta-analysis of randomised controlled trials. BMJ. 2021;372:m4573. 10.1136/bmj.m4573. 33441402 10.1136/bmj.m4573 PMC7804890

[10] Pop-Busui R, Januzzi JL, Bruemmer D, et al. Heart failure: an underappreciated complication of diabetes. A Consensus Report of the American Diabetes Association. Diabetes Care. 2022;45(7):1670–90. 10.2337/dci22-0014. 35796765 10.2337/dci22-0014 PMC9726978

[11] 2022 AHA/ACC/HFSA Guideline for the Management of Heart Failure: A Report of the American College of Cardiology/American Heart Association Joint Committee on Clinical Practice Guidelines | Circulation. Accessed November 10. 2023. https://www.ahajournals.org/doi/full/10.1161/CIR.0000000000001063 10.1161/CIR.0000000000001063 35363499

[12] Kidney Disease: Improving Global Outcomes (KDIGO) Diabetes Work Group. KDIGO 2022 Clinical Practice Guideline for Diabetes Management in chronic kidney disease. Kidney Int. 2022;102(5S):S1–127. 10.1016/j.kint.2022.06.008. 36272764 10.1016/j.kint.2022.06.008

[13] Yost O, DeJonckheere M, Stonebraker S, et al. Continuous glucose monitoring with low-carbohydrate Diet Coaching in adults with prediabetes: mixed methods pilot study. JMIR Diabetes. 2020;5(4):e21551. 10.2196/21551. 33325831 10.2196/21551 PMC7773517

[14] Aronson R, Brown RE, Chu L, et al. IMpact of flash glucose monitoring in pEople with type 2 diabetes inadequately controlled with non-insulin antihyperglycaemic ThErapy (IMMEDIATE): a randomized controlled trial. Diabetes Obes Metab. 2023;25(4):1024–31. 10.1111/dom.14949. 36546594 10.1111/dom.14949

[15] Evert AB, Dennison M, Gardner CD, et al. Nutrition Therapy for adults with diabetes or Prediabetes: a Consensus Report. Diabetes Care. 2019;42(5):731–54. 10.2337/dci19-0014. 31000505 10.2337/dci19-0014 PMC7011201

[16] Shin JI, Wang D, Fernandes G, et al. Trends in receipt of American Diabetes Association Guideline-recommended care among U.S. adults with diabetes: NHANES 2005–2018. Diabetes Care. 2021;44(6):1300–8. 10.2337/dc20-2541. 33863753 10.2337/dc20-2541 PMC8247496

[17] ElSayed NA, Aleppo G, Aroda VR, et al. 1. Improving Care and promoting health in populations: *standards of Care in Diabetes—2023*. Diabetes Care. 2023;46(Supplement1):S10–8. 10.2337/dc23-S001. 36507639 10.2337/dc23-S001 PMC9810463

[18] Davidson JA. The Increasing Role of Primary Care Physicians in Caring for Patients With Type 2 Diabetes Mellitus. *Mayo Clin Proc*. 2010;85(12 Suppl):S3-S4. 10.4065/mcp.2010.0466 10.4065/mcp.2010.0466 PMC2996164 21106869

[19] Chen JL, Krupp GR, Lo JY. The COVID-19 pandemic and changes in Health Care utilization among patients with type 2 diabetes. Diabetes Care. 2022;45(4):e74–6. 10.2337/dc21-2248. 35085378 10.2337/dc21-2248

[20] Worswick J, Wayne SC, Bennett R, et al. Improving quality of care for persons with diabetes: an overview of systematic reviews - what does the evidence tell us? Syst Rev. 2013;2:26. 10.1186/2046-4053-2-26. 23647654 10.1186/2046-4053-2-26 PMC3667096

[21] Share DA, Campbell DA, Birkmeyer N, et al. How a Regional Collaborative of hospitals and Physicians in Michigan Cut costs and improved the quality of care. Health Aff (Millwood). 2011;30(4):636–45. 10.1377/hlthaff.2010.0526. 21471484 10.1377/hlthaff.2010.0526

[22] Collaborative Quality Initiatives. – Value Partnerships.com — Blue Cross Blue Shield of Michigan. https://www.valuepartnerships.com/programs/collaborative-quality-initiatives/

[23] ElSayed NA, Aleppo G, Aroda VR, et al. 11. Chronic kidney Disease and Risk Management: *standards of Care in Diabetes—2023*. Diabetes Care. 2023;46(Supplement1):S191–202. 10.2337/dc23-S011. 36507634 10.2337/dc23-S011 PMC9810467

[24] ElSayed NA, Aleppo G, Aroda VR, et al. 10. Cardiovascular Disease and Risk Management: *standards of Care in Diabetes—2023*. Diabetes Care. 2023;46(Supplement1):S158–90. 10.2337/dc23-S010. 36507632 10.2337/dc23-S010 PMC9810475

[25] Arnott C, Li Q, Kang A, et al. Sodium-glucose cotransporter 2 inhibition for the Prevention of Cardiovascular events in patients with type 2 diabetes Mellitus: a systematic review and Meta‐analysis. J Am Heart Assoc. 2020;9(3):e014908. 10.1161/JAHA.119.014908. 31992158 10.1161/JAHA.119.014908 PMC7033896

[26] Bhatt DL, Szarek M, Steg PG, et al. Sotagliflozin in patients with diabetes and recent worsening heart failure. N Engl J Med. 2021;384(2):117–28. 10.1056/NEJMoa2030183. 33200892 10.1056/NEJMoa2030183

[27] Bhatt DL, Szarek M, Pitt B, et al. Sotagliflozin in patients with diabetes and chronic kidney disease. N Engl J Med. 2021;384(2):129–39. 10.1056/NEJMoa2030186. 33200891 10.1056/NEJMoa2030186

[28] American Diabetes Association. 8. Pharmacologic approaches to Glycemic Treatment: *standards of Medical Care in Diabetes—2018*. Diabetes Care. 2018;41(Supplement1):S73–85. 10.2337/dc18-S008. 29222379 10.2337/dc18-S008

[29] Heidenreich PA, Bozkurt B, Aguilar D, et al. 2022 AHA/ACC/HFSA Guideline for the management of Heart failure: a report of the American College of Cardiology/American Heart Association Joint Committee on Clinical Practice guidelines. Circulation. 2022;145(18):e895–1032. 10.1161/CIR.0000000000001063. 35363499 10.1161/CIR.0000000000001063

[30] Fonseca V, McDuffie R, Calles J, et al. Determinants of Weight Gain in the action to Control Cardiovascular Risk in Diabetes Trial. Diabetes Care. 2013;36(8):2162–8. 10.2337/dc12-1391. 23412077 10.2337/dc12-1391 PMC3714487

[31] Sharma SP, Russo A, Deering T, Fisher J, Lakkireddy D. Prior authorization. JACC Clin Electrophysiol. 2020;6(6):747–50. 10.1016/j.jacep.2020.04.022. 32553230 10.1016/j.jacep.2020.04.022

[32] Gao Y, Peterson E, Pagidipati N. Barriers to prescribing glucose-lowering therapies with cardiometabolic benefits. Am Heart J. 2020;224:47–53. 10.1016/j.ahj.2020.03.017. 32304879 10.1016/j.ahj.2020.03.017

[33] Isajev N, Bjegovic-Mikanovic V, Bukumiric Z, Vrhovac D, Lalic NM. Predictors of clinical inertia and type 2 diabetes: Assessment of Primary Care Physicians and their patients. Int J Environ Res Public Health. 2022;19(8):4436. 10.3390/ijerph19084436. 35457303 10.3390/ijerph19084436 PMC9031531

[34] Bibeau WS, Fu H, Taylor AD, Kwan AYM. Impact of out-of-Pocket Pharmacy costs on branded medication adherence among patients with type 2 diabetes. J Manag Care Spec Pharm. 2016;22(11):1338–47. 10.18553/jmcp.2016.22.11.1338. 27783549 10.18553/jmcp.2016.22.11.1338 PMC10397590

[35] Santos Cavaiola T, Kiriakov Y, Reid T. Primary Care management of patients with type 2 diabetes: overcoming Inertia and advancing Therapy with the Use of Injectables. Clin Ther. 2019;41(2):352–67. 10.1016/j.clinthera.2018.11.015. 30655008 10.1016/j.clinthera.2018.11.015

[36] Rea F, Ciardullo S, Savaré L, Perseghin G, Corrao G. Comparing medication persistence among patients with type 2 diabetes using sodium-glucose cotransporter 2 inhibitors or glucagon-like peptide-1 receptor agonists in real-world setting. Diabetes Res Clin Pract. 2021;180:109035. 10.1016/j.diabres.2021.109035. 34487757 10.1016/j.diabres.2021.109035

[37] Gill GS, Latif A, Hilleman D, Lavie CJ, Alla VM. Challenges in implementing evidence based cross-disciplinary therapies: are Cardiovascular specialists ready to claim SGLT-2 inhibitors and GLP-1 analogs? Curr Probl Cardiol. 2022;47(7):100878. 10.1016/j.cpcardiol.2021.100878. 34078543 10.1016/j.cpcardiol.2021.100878

[38] Beck RW, Riddlesworth TD, Ruedy K, et al. Continuous glucose monitoring Versus Usual Care in patients with type 2 diabetes receiving multiple daily insulin injections: a Randomized Trial. Ann Intern Med. 2017;167(6):365. 10.7326/M16-2855. 28828487 10.7326/M16-2855

[39] Martens T, Beck RW, Bailey R, et al. Effect of continuous glucose monitoring on Glycemic Control in patients with type 2 diabetes treated with basal insulin: a Randomized Clinical Trial. JAMA. 2021;325(22):2262. 10.1001/jama.2021.7444. 34077499 10.1001/jama.2021.7444 PMC8173473

[40] ElSayed NA, Aleppo G, Aroda VR et al. 7. Diabetes Technology: *Standards of Care in Diabetes* —. 2023. *Diabetes Care*. 2023;46(Supplement_1):S111-S127. 10.2337/dc23-S007 10.2337/dc23-S007 PMC9810474 36507635

[41] Oser TK, Hall TL, Dickinson LM, et al. Continuous glucose monitoring in primary care: understanding and supporting clinicians’ use to Enhance Diabetes Care. Ann Fam Med. 2022;20(6):541–7. 10.1370/afm.2876. 36443083 10.1370/afm.2876 PMC9705045

[42] Sainsbury E, Kizirian NV, Partridge SR, Gill T, Colagiuri S, Gibson AA. Effect of dietary carbohydrate restriction on glycemic control in adults with diabetes: a systematic review and meta-analysis. Diabetes Res Clin Pract. 2018;139:239–52. 10.1016/j.diabres.2018.02.026. 29522789 10.1016/j.diabres.2018.02.026

[43] Westman EC, Tondt J, Maguire E, Yancy WS. Implementing a low-carbohydrate, ketogenic diet to manage type 2 diabetes mellitus. Expert Rev Endocrinol Metab. 2018;13(5):263–72. 10.1080/17446651.2018.1523713. 30289048 10.1080/17446651.2018.1523713

[44] Yancy WS, Crowley MJ, Dar MS, et al. Comparison of Group Medical visits combined with Intensive Weight Management vs Group Medical visits alone for glycemia in patients with type 2 diabetes: a Noninferiority Randomized Clinical Trial. JAMA Intern Med. 2020;180(1):70. 10.1001/jamainternmed.2019.4802. 31682682 10.1001/jamainternmed.2019.4802 PMC6830502

[45] Athinarayanan SJ, Adams RN, Hallberg SJ, et al. Long-Term effects of a Novel continuous remote care intervention including nutritional ketosis for the management of type 2 diabetes: a 2-Year non-randomized clinical trial. Front Endocrinol. 2019;10:348. 10.3389/fendo.2019.00348. 10.3389/fendo.2019.00348 PMC6561315 31231311

[46] ElSayed NA, Aleppo G, Aroda VR, et al. 5. Facilitating Positive Health Behaviors and Well-being to Improve Health outcomes: *standards of Care in Diabetes—2023*. Diabetes Care. 2023;46(Supplement1):S68–96. 10.2337/dc23-S005. 36507648 10.2337/dc23-S005 PMC9810478

[47] Griauzde DH, Ling G, Wray D, et al. Continuous glucose monitoring with low-Carbohydrate Nutritional Coaching to improve type 2 Diabetes Control: Randomized Quality Improvement Program. J Med Internet Res. 2022;24(2):e31184. 10.2196/31184. 35107429 10.2196/31184 PMC8851329

[48] McArdle PD, Greenfield SM, Avery A, Adams GG, Gill PS. Dietitians’ practice in giving carbohydrate advice in the management of type 2 diabetes: a mixed methods study. J Hum Nutr Diet. 2017;30(3):385–93. 10.1111/jhn.12436. 28276183 10.1111/jhn.12436

[49] Cucuzzella M, Riley K, Isaacs D. Adapting medication for type 2 diabetes to a low Carbohydrate Diet. Front Nutr. 2021;8:688540. 10.3389/fnut.2021.688540. 34434951 10.3389/fnut.2021.688540 PMC8380766

[50] About Learning Health Systems. Accessed July 14. 2023. https://www.ahrq.gov/learning-health-systems/about.html

[51] Carpenter D, Hassell S, Mardon R, et al. Using Learning communities to support adoption of Health Care innovations. Jt Comm J Qual Patient Saf. 2018;44(10):566–73. 10.1016/j.jcjq.2018.03.010. 30064957 10.1016/j.jcjq.2018.03.010

[52] Collaboratives MIHINMIHIN. PPQC. Accessed November 24, 2023. https://mihin.org/collaboratives/

[53] Chin MH. Quality improvement implementation and disparities: the case of the health disparities collaboratives. Med Care. 2010;48(8):668–75. 10.1097/MLR.0b013e3181e3585c. 20613665 10.1097/MLR.0b013e3181e3585c PMC3401560

[54] Hill-Briggs F, Adler NE, Berkowitz SA, et al. Social Determinants of Health and Diabetes: A Scientific Review. Diabetes Care. 2021;44(1):258–79. 10.2337/dci20-0053. 10.2337/dci20-0053 PMC7783927 33139407

[55] WELCOME TO JUMPSTART:A low carb lifestyle for Type 2 Diabetes. 2022. Accessed October 26, 2023. https://jumpstart.mct2d.org/

[56] Damschroder LJ, Reardon CM, Widerquist MAO, Lowery J. The updated Consolidated Framework for Implementation Research based on user feedback. Implement Sci. 2022;17(1):75. 10.1186/s13012-022-01245-0. 36309746 10.1186/s13012-022-01245-0 PMC9617234

[57] Hamilton AB, Finley EP. Qualitative methods in implementation research: an introduction. Psychiatry Res. 2019;280:112516. 10.1016/j.psychres.2019.112516. 31437661 10.1016/j.psychres.2019.112516 PMC7023962

[58] SAS Institute Inc. SAS/ACCESS^®^ 9.4 Interface to ADABAS: Reference. Cary, NC: SAS Institute Inc; 2013.

[59] Britto MT, Fuller SC, Kaplan HC, et al. Using a network organisational architecture to support the development of Learning Healthcare systems. BMJ Qual Saf. 2018;27(11):937–46. 10.1136/bmjqs-2017-007219. 29438072 10.1136/bmjqs-2017-007219 PMC6225794

