# Supplementary material for: The Michigan Collaborative for Type 2 Diabetes (MCT2D): Development and implementation of a statewide collaborative quality initiative
Source: BMC Health Serv Res. 2024 Oct 17;24:1254. doi: 10.1186/s12913-024-11520-z (PMC11487891; doi:10.1186/s12913-024-11520-z)
Supplement: Supplementary file 1 — Supplementary Material 1: Appendix 1 [file 12913_2024_11520_MOESM1_ESM.pdf]

MCT2D PO Scorecard

Michigan Collaborative for Type 2 Diabetes (MCT2D) CQI PO Scorecard				
Measurement Period: 09/01/2023 - 08/31/2024<br>PO reward dates: January 2025, July 2025				
**Category**	**Measure #**	**Critera**	**Expectation**	**Points**
**Meeting Attendance**	**1**	Attend at least 7 out of 9 PO Monthly Calls.	MCT2D hosts monthly calls with two different attendance options all months except for April, June, and October during regional meetings and collaborative wide meetings. If calls are canceled on an ad hoc basis, adjustments will be made to the attendance requirement. One participant per PO is required.<br><br>5-6 meetings will earn 4 points, 3-4 meetings will earn 2 points, 0-2 meetings will earn 0 points.	5
	**2**	Meet with other PO leadership who have chosen the same quality initiative focus for Year 3 VBR.	MCT2D is offering three separate quality initiative (QI) focuses based off the MCT2D pillars- increasing use of low carbohydrate diet, supporting guideline directed medication prescribing, and increasing prescribing of continuous glucose monitors (CGMs). Per the value-based reimbursement, each PO will choose an initiative to focus on. MCT2D will organize and convene a separate meeting for each QI focus and POs will be expected to join one of these meetings.	5
	**3**	Attend annual check in meeting.	The administrative lead and/or primary contact from the physician organizations participates in an annual check in call with the MCT2D coordinating center.	10
	**4**	Attend collaborative wide meeting.	An administrative lead and clinical champion from the physician organization attend the annual June collaborative wide meeting.	5
**Data**	**5**	Ensure completeness of data- BMI.	MCT2D completed a PO-level evaluation of submission rates of body mass index (BMI) in the Provider-Payor Quality Collaborative (PPQC) data. We calculated the quartiles for submission and have provided each PO their current submission rate. We will be requiring that each PO reach the next quartile for submission, with the maximum rate for BMI submission being 95%. MCT2D will report on the Administrative Portal your current submission rate and target rate, as well as provide updates with each data dashboard refresh.	5
	**6**	Ensure completeness of data- A1C.	MCT2D completed a PO-level evaluation of submission rates of hemoglobin A1C (A1C) in the PPQC data. We will be requiring that each PO reach the next quartile for submission, with the maximum rate for A1C submission being 82%. MCT2D will report on the Administrative Portal your current submission rate and target rate, as well as provide updates with each data dashboard refresh.	5
	**7**	Submit additional data elements.	As requested by the MCT2D Coordinating Center, develop a plan and begin submitting additional data elements, e.g., serum creatinine or unit measures for weight. If POs cannot send the requested data element currently or develop a plan to include this data in the near-term future, submit an explanation to MCT2D with the barriers to submitting.	5
**Contacts**	**8**	Maintain physician organization contacts.	Ensure that an administrative lead, clinical champion, and data contact are identified and listed in the administrative portal. MCT2D will notify you if this information is missing or if we receive an email bounce-back. POs will have one month to identify a replacement role.	5
	**9**	Designate separate clinical champions each practice.	Designating a separate clinical champion* for each practice instead of having one person fill this role for multiple practices. If >95% of practices in your integrated health system are enrolled in MCT2D, the coordinating center will work with you to develop an individualized plan.	10
	**10**	Maintain practice level contacts.	Ensure that the clinical champion role and practice liaison role are identified and listed in the administrative portal, and that contact information is kept current and complete.MCT2D will notify you if this information is missing or if we receive an email bounce-back. POs will have one month to identify a replacement role.	5
**Engagement**	**11**	Communicate with practices to ensure regional meeting registration.	Monitors practice level clinical champion registration status for regional meetings and proactively reaches out to ones who have not registered as the meeting nears to ensure that they or a substitute is registered for, and attends the meeting. This metric will be considered met if the PO's communications result in all clinical champions/substitutes being registered for meeting two weeks prior to the meeting date and/or the PO directly informs MCT2D if any practices cannot send an attendee.	5
	**12**	Meet PO level learning community requirement.	Complete one of the activities listed under the PO Level VBR Options: having someone from your PO or your practices present on an MCT2D panel, participate in an interview for a blog post, have a representative from your organization participate on the steering committee, or refer a patient to MCT2D to share their story or serve on the patient advisory committee. Additional opportunities may be available throughout the year that MCT2D will make you aware of.	15
	**13**	Support practices in quality improvement work on guideline directed medication prescribing, low carb diet, or continuous glucose monitors.	For POs who chose guideline directed medication prescribing or CGMs, determine a PO level goal for medications and CGMs, convene meetings with participating practices and support them in developing an improvement plan, and report progress and challenges/successes in meeting these goals to MCT2D. For POs who chose low carbohydrate: work with the coordinating center to develop a patient reported outcomes goal and practice changes based on results. Attending required meetings and submitting required documentation will meet the criteria to earn these points. Refer to the Year 3 PCP VBR measures for more detail.	20
			**Total Points**	**100**
