# Supplementary material for: The Michigan Collaborative for Type 2 Diabetes (MCT2D): Development and implementation of a statewide collaborative quality initiative
Source: BMC Health Serv Res. 2024 Oct 17;24:1254. doi: 10.1186/s12913-024-11520-z (PMC11487891; doi:10.1186/s12913-024-11520-z)
Supplement: Supplementary file 2 — Supplementary Material 2: Appendix 2 [file 12913_2024_11520_MOESM2_ESM.pdf]

Resources for Getting Started with

Continuous Glucose Monitors

MCT2D
MICHIGAN COLLABORATIVE FOR TYPE 2 DIABETES

Continuous glucose monitors (CGMs) can help you manage your diabetes by providing real-time glucose readings, eliminating the need for frequent finger pokes. CGMs allow you to learn how food impacts your blood sugar, helping you make healthier decisions in the future. You can also easily share glucose data with your family and healthcare team while tracking your trends over time. We've gathered some resources to help you get started with your CGM. If you have any more questions, reach out to your healthcare team for support.

Two of the most common brands of CGMs used by patients with type 2 diabetes:

ABBOTT

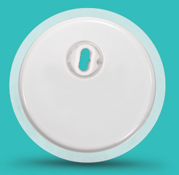

FreeStyle Libre 2 or 3
from Abbott

DEXCOM

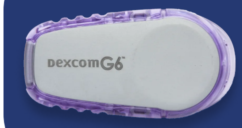

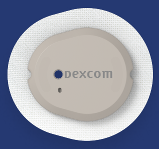

G6 or G7
from Dexcom

I am using or was prescribed this brand and model of CGM: ______________________
Write in your CGM here

I want to learn more about getting started with a CGM.

ABBOTT

FreeStyle Libre 2 or 3
Get the Support you need with guides and videos.

FreeStyle Libre 3: ***www.freestyle.abbott/us-en/freestyle-libre-3-resources.html***
FreeStyle Libre 2: ***www.free style.abbott/us-en/freestyle-libre-2-resources.html***

DEXCOM

Dexcom G6 or G7
Attend a free online classes on a variety of topics including app and receiver setup, CGM basics, and data sharing. See back for more info.

Register online at www.dexcom.com/en-us/dexcom-care

I need help affording my CGM.

ABBOTT

FreeStyle Libre 2 or 3*
If you have a commercial insurance plan with a high deductible, call the Abbott customer care team at ***(844) 330-5535 or scan the QR code*** to ask about two voucher options you may be eligible for:

1. Receive 1 free sensor (14 days)
2. Receive 2 sensors per month for $75 monthly copay *(Talk to your local pharmacist for more information)*

* If you have Medicare, the Libre 3 is only available at medical suppliers at this time.

DEXCOM

Dexcom G6 or G7
Have Dexcom complete an insurance benefits check and get help understanding coverage by completing a new patient form at ***www.dexcom.com/en-us/start***

If you have a commercial insurance plan with a high deductible, visit ***www.dexcom.com/en-us/savings-center or scan the QR code***

I need technical support or a replacement sensor.

ABBOTT

FreeStyle Libre 2 or 3
Call the Abbott customer care team at ***(855) 632-8658*** or complete an online form by scanning the QR code.

www.freestyle.abbott/us-en/support/contact-us.html

DEXCOM

Dexcom G6 or G7
Call the Dexcom product support line at **(844) 607-8398** or complete an online form at **www.dexcom.com/en-us/contact**

Tip: Keep your CGM's packaging incase you need to request a replacement.

I want more education and support as I start using my CGM.

ABBOTT

FreeStyle Libre 2 or 3
The MyFreeStyle program is a free 6+ month diabetes education and support program that will teach you how to use your CGM to manage your diabetes. Visit:

www.StartMyFreeStyle.com

DEXCOM

Dexcom G6 or G7
Dexcom offers robust online education for getting started and using a G6 or G7 CGM including videos and live chat support. Visit:

www.dexcom.com *(then click 'Learn')*

Additional information on Dexcom learning opportunities in Michigan

DEXCOM

Online Classes for G6 or G7 *(Register online at www.dexcom.com/en-us/dexcom-care)*

- **What a Dexcom G7 Can Do For You**
  An introduction to the G7 CGM, the benefits of trend arrows, alerts, and advantages of CGM vs. fingersticks with a blood glucose monitor. You will review insights gained from viewing CGM readings in Clarity and how it can help you better manage your diabetes.
  This class will not provide step by step instruction on set up.

- **G7 Getting Started *(separate classes for the G7 app and the G7 receiver)***
  Walk through setting up the G7 CGM for the first time, using either the G7 app on a smart phone or the G7 receiver. Other training topics will include how to change the sensor every 10 days, trend arrows, guidelines to follow when using the G7 system for treatment decisions and how the alerts work.

- **Clarity: Preparing for Your HCP Appointment with G6 and G7**
  Review Dexcom Clarity including how to access Clarity, how data is imported into Clarity and how to share reports with your healthcare team. The class will provide an explanation of Clarity reports and discuss insights that can be gained to help facilitate a more meaningful discussion with your healthcare team.

Scan the QR code to register for a Dexcom online class.

For any additional Dexcom training needed, please call 1-888-738-3646 and choose "training and education" as the prompt.

MCT2D
www.MCT2D.org

Clinician Decision Aid

SGLT2is / GLP-1 RAs for Type 2 Diabetes

MCT2D MICHIGAN COLLABORATIVE FOR TYPE 2 DIABETES

SGLT2 inhibitors and GLP-1 receptor agonists are first-line treatments for T2D in patients with cardiorenal disease. ***This aid is meant to be used alongside your own clinical judgment, guideline-directed therapy, and prescribing information to guide individualization of diabetes treatment.***[1]

ALL PATIENTS WITH T2D — Recommend lifestyle change with reduced carb intake and weight loss if indicated

CLINICAL GOAL — To reduce cardiorenal risk independent of A1C in high risk patients

- **Established ASCVD**[b]
  - **GLP-1 RA or SGLT2i Shared Decision Making:**
    - Dulaglutide
    - Liraglutide
    - Semaglutide SQ
    - or
    - Canagliflozin
    - Empagliflozin
  - If A1C above target, consider adding SGLT2i to GLP-1 RA and vice versa.
- **Heart Failure**
  - **HFpEF**[d]: Dapagliflozin, Empagliflozin; *(off label)* Canagliflozin[f], Ertugliflozin[f]
  - **HFrEF**[d]: Dapagliflozin, Empagliflozin; *(off label)* Canagliflozin[f], Ertugliflozin[f]
- **CKD**[c]
  - **CKD**[c]: Dapagliflozin[e], Empagliflozin[e], Canagliflozin
  - If A1C above target, consider adding SGLT2i to GLP-1 RA and vice versa.

CLINICAL GOAL — Achieve glycemic control[a] and promote weight loss

- **Set weight loss goals and engage in shared decision making for therapeutic interventions**
  - **GLP-1 RA/GIP Medications for T2D with efficacy for weight loss:**
    - Very high: Semaglutide SQ, Tirzepatide
    - High: Dulaglutide, Liraglutide

Legend:
- ***FDA Labeled*** *(preferred)* For T2D & cardiorenal risk factors
- ***Off label*** *(see footnote f)*

Consider Metformin with above agents for additive glycemic lowering

Footnotes

a. **Glycemic lowering as primary clinical goal:** Use Metformin or in combination with other T2D medications to achieve patient's glycemic goal. The following glycemic lowering categories (modified from ADA Standards of Care[1])
   i. **VERY HIGH:** dulaglutide (high dose), semaglutide, tirzepatide
   ii. **HIGH:** GLP-1 RA (not previously listed, metformin, SGLT2i, sulfonylurea, TZD
   iii. **INTERMEDIATE:** DPP-4i

b. **Consider use in HIGH risk patients:** ADA Standards gives weaker recommendation for use given CVOT data were not powered for primary analysis in this subgroup. This subgroup includes: age $\geq$ 55 with 2 or more risk factors (e.g, obesity, hypertension, smoking, hyperlipidemia, albuminuria).

c. **Diagnosis of CKD (eGFR<60) and/or presence of albuminuria:** Determine after repeating measures of GFR and albuminuria twice, 3 months apart.

d. **HFpEF:** Heart failure with preserved ejection fraction. **HFrEF:** Heart failure with reduced ejection fraction (left ventricular EF $\leq$ 40%).

e. **When using for HF and/or CKD protection:** Irrespective of T2D diagnosis, dose is 10mg daily, and can be used at renal functions below renal cutoffs for glycemic lowering.

f. **Canagliflozin/Ertugliflozin in HF:** Data for use from secondary outcomes of CVOT T2D trials.

See back for list of generic and brand drug names

MCT2D Medication Coverage Guide

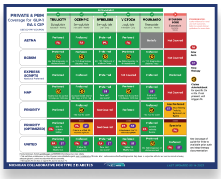

michmed.org/47nKe

Prescribing Reminders and Recommendations

GLP-1 RA

1. **Contraindications:** Personal or family hx medullary thyroid cancer or MEN-2-syndrome, pregnancy, lactation, or allergy to medication.
2. **Precautions:** History of pancreatitis, severe GI disease (gastroparesis), gallbladder disease. In patients with impaired renal function and reporting severe gastrointestinal side effects, closely monitor renal function.
3. **Assess baseline:** Gastrointestinal status for nausea, vomiting, and diarrhea.
4. **Medication adjustment:** If A1c less than 9%, consider lowering basal insulin by 10% and prandial insulin by 30-40%.[2]
5. **Key counseling points:** Counsel patients on mitigating strategies for common GI side effects and expected improvement over time.
6. **Dose titration:** Provide clear dose titration instructions when prescribing agents that have sensitizing starting dose.
7. **Combination therapy:** Avoid combination with DPP4-inhibitors.
8. **Prior to surgery:** Hold day prior or day of surgery or week prior if on weekly GLP-1RA. See Reference 3 for more details.

Generic and Brand Drug Names

GLP-1 RAs Generic and Brand Names	
Dulaglutide	Trulicity
Exenatide	Byetta
Exenatide XR	Bydureon BCise
Liraglutide	Victoza
Semaglutide SQ	Ozempic
Semaglutide (oral)	Rybelsus

SGLT2is Generic and Brand Names	
Canagliflozin	Invokana
Dapagliflozin	Farxiga
Empagliflozin	Jardiance
Ertugliflozin	Steglatro

Patient GLP-1 RA & SGLT2i Handouts

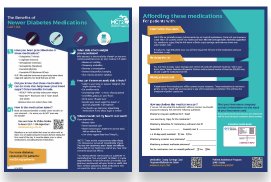

michmed.org/bmx5B

SGLT2i

1. **Contraindications:** T1D, ESRD, dialysis, pregnancy, lactation, allergy to medication, or very low carbohydrate diet (less than 50g/day).
2. **Renal function:** Prior to prescribing, review recent eGFR to ensure eligibility for glycemic lowering based on renal cutoffs.
3. **Amputation risk:** Prior to prescribing, consider risk versus benefit in patients with a history of PAD, recent foot infections, or history of foot amputations.
4. **Consider alternative:** If A1c greater than 10%, to avoid excess glucosuria – consider another agent to lower glucose, prior to starting SGLT2i.
5. **Medication adjustment, if A1C less than 8.5% and on:**
   a. ***Insulin*** – Consider reducing total daily dose of insulin by 10-20%. ***Caution: Avoid insulin discontinuation to minimize risk of euglycemic DKA.***
   b. ***Sulfonylurea*** – Discontinue or lower dose by 50%.
   c. ***If A1C greater than 8.5%*** – monitor closely and adjust other medications as needed.[4]
6. **Key counseling points:** Counsel patients on common side effects, importance of hydration, sick day management, notification of healthcare team if adopting a lower carbohydrate diet (less than 130g/day) and euglycemic DKA. Patients should be reminded to check feet daily, and notify prescriber immediately if any new onset foot pain, soreness, or ulcers.
7. **Prior to surgery:** Hold medication for at least 4 days if procedure is associated with prolonged fasting.
8. **Follow-up:** If patient is high-risk, complex, and/or has impaired renal function, consider 4-week follow-up. Repeat metabolic panel and dose adjust as needed. When considering dose increase, balance glycemic lowering benefit with risk of dose intolerance (hypotension, increased urinary frequency, urinary/mycotic infections, and volume status).

References

1. *American Diabetes Association. 9. Pharmacologic Approaches to Glycemic Treatment: Standards of Medical Care in Diabetes-2023. Clin Diabetes 2023;41(1):4–31. doi.org/10.2337/cd23-as01*
2. *Sassenrath K, Phillips BB, Stone RH. Evaluation of GLP-1 Receptor Agonists in Combination With Multiple Daily Insulin Injections for Type 2 Diabetes. J Pharm Pract. 2022 Dec;35(6):979-990. doi: 10.1177/08971900211010678. Epub 2021 Apr 29. PMID: 33926305.*
3. *Joshi GP, Abdelmalak BB, Weigel WA, et al. American Society of Anesthesiologists Consensus-Based Guidance on Preoperative Management of Patients (Adults and Children) on Glucagon-Like Peptide-1 (GLP-1) Receptor Agonists. American Society of Anesthesiologists. 2023 Jun 29. michmed.org/JQmKJ*
4. *Gomez-Peralta F, Abreu C, Lecube A, et al. Practical Approach to Initiating SGLT2 Inhibitors in Type 2 Diabetes [published correction appears in Diabetes Ther. 2017 Aug 23;:]. Diabetes Ther. 2017;8(5):953-962. doi:10.1007/s13300-017-0277-0*

Updated Sept 2023
By Heidi L. Diez, PharmD, BCACP

WEEKLY CARB & BLOOD SUGAR TRACKER

NAME: ____________________

DOB: ____________________

TARGET RANGES

PRE-MEAL	POST-MEAL

OPTIONAL GOALS
(Complete at beginning of week)

Daily carb intake:

Minutes of physical activity:

Daily blood sugar checks:

REFLECTIONS
(Complete at end of week)

This week my energy level was: (circle)

1 2 3 4 5

Low I felt very tired — **High** I had lots of energy

This week my food cravings were: (circle)

1 2 3 4 5

Low I had no cravings — **High** I had lots of cravings

NOTES:

Always follow your healthcare provider's instructions on when and how often to check your blood sugar.

MCT2D MICHIGAN COLLABORATIVE FOR TYPE 2 DIABETES

MCT2D.org

MONDAY DATE:

BREAKFAST :

Blood sugar **before** meal:

FOOD / DRINK ITEM	CARBS

Blood sugar **2 hours** after meal:

LUNCH :

Blood sugar **before** meal:

FOOD / DRINK ITEM	CARBS

Blood sugar **2 hours** after meal:

DINNER :

Blood sugar **before** meal:

FOOD / DRINK ITEM	CARBS

Blood sugar **2 hours** after meal:

SNACK :

FOOD / DRINK ITEM	CARBS

Blood sugar **before bed**:

Total carbs for the day:

PHYSICAL ACTIVITY	DURATION

How do you feel about the meals you ate today? Good Neutral Needs work

TUESDAY DATE:

BREAKFAST :

Blood sugar **before** meal:

FOOD / DRINK ITEM	CARBS

Blood sugar **2 hours** after meal:

LUNCH :

Blood sugar **before** meal:

FOOD / DRINK ITEM	CARBS

Blood sugar **2 hours** after meal:

DINNER :

Blood sugar **before** meal:

FOOD / DRINK ITEM	CARBS

Blood sugar **2 hours** after meal:

SNACK :

FOOD / DRINK ITEM	CARBS

Blood sugar **before bed**:

Total carbs for the day:

PHYSICAL ACTIVITY	DURATION

How do you feel about the meals you ate today? Good Neutral Needs work

WEDNESDAY DATE:

BREAKFAST :

Blood sugar **before** meal:

FOOD / DRINK ITEM	CARBS

Blood sugar **2 hours** after meal:

LUNCH :

Blood sugar **before** meal:

FOOD / DRINK ITEM	CARBS

Blood sugar **2 hours** after meal:

DINNER :

Blood sugar **before** meal:

FOOD / DRINK ITEM	CARBS

Blood sugar **2 hours** after meal:

SNACK :

FOOD / DRINK ITEM	CARBS

Blood sugar **before bed**:

Total carbs for the day:

PHYSICAL ACTIVITY	DURATION

How do you feel about the meals you ate today? Good Neutral Needs work

THURSDAY
DATE:

BREAKFAST
:

Blood sugar **before** meal:

FOOD / DRINK ITEM	CARBS

Blood sugar **2 hours** after meal:

LUNCH
:

Blood sugar **before** meal:

FOOD / DRINK ITEM	CARBS

Blood sugar **2 hours** after meal:

DINNER
:

Blood sugar **before** meal:

FOOD / DRINK ITEM	CARBS

Blood sugar **2 hours** after meal:

SNACK
:

FOOD / DRINK ITEM	CARBS

Blood sugar **before bed**:

Total carbs for the day:

PHYSICAL ACTIVITY	DURATION

How do you feel about the meals you ate today? Good Neutral Needs work

FRIDAY
DATE:

BREAKFAST
:

Blood sugar **before** meal:

FOOD / DRINK ITEM	CARBS

Blood sugar **2 hours** after meal:

LUNCH
:

Blood sugar **before** meal:

FOOD / DRINK ITEM	CARBS

Blood sugar **2 hours** after meal:

DINNER
:

Blood sugar **before** meal:

FOOD / DRINK ITEM	CARBS

Blood sugar **2 hours** after meal:

SNACK
:

FOOD / DRINK ITEM	CARBS

Blood sugar **before bed**:

Total carbs for the day:

PHYSICAL ACTIVITY	DURATION

How do you feel about the meals you ate today? Good Neutral Needs work

SATURDAY
DATE:

BREAKFAST
:

Blood sugar **before** meal:

FOOD / DRINK ITEM	CARBS

Blood sugar **2 hours** after meal:

LUNCH
:

Blood sugar **before** meal:

FOOD / DRINK ITEM	CARBS

Blood sugar **2 hours** after meal:

DINNER
:

Blood sugar **before** meal:

FOOD / DRINK ITEM	CARBS

Blood sugar **2 hours** after meal:

SNACK
:

FOOD / DRINK ITEM	CARBS

Blood sugar **before bed**:

Total carbs for the day:

PHYSICAL ACTIVITY	DURATION

How do you feel about the meals you ate today? Good Neutral Needs work

SUNDAY
DATE:

BREAKFAST
:

Blood sugar **before** meal:

FOOD / DRINK ITEM	CARBS

Blood sugar **2 hours** after meal:

LUNCH
:

Blood sugar **before** meal:

FOOD / DRINK ITEM	CARBS

Blood sugar **2 hours** after meal:

DINNER
:

Blood sugar **before** meal:

FOOD / DRINK ITEM	CARBS

Blood sugar **2 hours** after meal:

SNACK
:

FOOD / DRINK ITEM	CARBS

Blood sugar **before bed**:

Total carbs for the day:

PHYSICAL ACTIVITY	DURATION

How do you feel about the meals you ate today? Good Neutral Needs work

LOW CARB CHEATSHEET

0g CARB FOODS (Per serving)

Meats
Beef, Lamb, Pork, Veal (3 oz)

Poultry
Chicken, Turkey, Duck (3 oz)

Fish / Shellfish
Salmon, Tuna, Sardines, Shrimp, Lobster, Crab (3 oz)

Eggs
whole or whites (1 egg)

Lemon, Lime, Vinegar, Herbs, Spices (1 tsp)

Fats: Oils
Avocado, Olive, Sesame, Peanut, Canola (1 tsp)

Fats: Other
Butter (1 tsp), Mayo (1 tbsp), Heavy cream (1 tbsp)

Beverages
Water, Black coffee, Unsweetened tea, sparkling water (8 oz)

1-5g CARB FOODS (Per serving)

Leafy Greens
Lettuce, Spinach, Arugula, Kale, Collards, Turnip, Romaine (1 cup raw or ½ cup cooked)

Non-starchy Vegetables
Asparagus, Bell peppers, Bok Choy, Brussel Sprouts, Cucumbers, Cabbage, Cauliflower, Eggplant, Onions (1 cup raw or ½ cup cooked)

Avocado
(1 oz or 2 tbsp)

Deli Meat & Bacon
(1 oz or 2 slices)

Dairy
Cheese (1 oz or ¼ cup shredded), Cream cheese (1 tbsp)

Tofu
(3 oz)

Hummus
(1 tbsp)

5-10g CARB FOODS (Per serving)

Edamame
(1/2 cup peeled)

Beets
(1/2 cup)

Raspberries
(1/2 cup or 2 oz)

Strawberries
(6 medium or 2 oz)

Carrots
(10 baby carrots or ¾ cup)

Squash
butternut, Spaghetti, Acorn, Pumpkin (1 cup)

Nuts
Walnuts, Almonds, Brazil Nuts, Cashews, Pistachios, Peanuts (1 oz)

Seeds
Flaxseed (3 tbsp), Sunflower (1 tbsp) Chia Seeds (2 tbsp)

Full-fat plain Greek yogurt
(1 cup)

10-20g CARB FOODS (Per serving)

Cow's milk
(1 cup)

Blueberries, Blackberries
(1/2 cup or 2.5 oz)

Beans, Lentils, Quinoa
(1/2 cup cooked)

Sweet Potato
(1/2 cup mashed)

Kiwi, Peach
(1 whole)

Rolled Oats
(1/3 cup)

Melon / Watermelon
(1 cup diced)

Green Peas, Corn
(1/2 cup)

HIGH CARB FOODS

Brown Rice
(50g per 1 cup cooked)

Fruit Smoothie
(35g per 8 oz)

100% Juice
(26-30g per 8 oz)

Whole Wheat Bread
(15-20g per slice)

Sugary Cereal
(30g per 1 cup with no milk)

Fruit Jam or Jelly
(30g per 2 tbsp)

10" Tortilla
(40g per wrap)
