# Supplementary material for: The Michigan Collaborative for Type 2 Diabetes (MCT2D): Development and implementation of a statewide collaborative quality initiative
Source: BMC Health Serv Res. 2024 Oct 17;24:1254. doi: 10.1186/s12913-024-11520-z (PMC11487891; doi:10.1186/s12913-024-11520-z)
Supplement: Supplementary file 3 — Supplementary Material 3: Appendix 3 [file 12913_2024_11520_MOESM3_ESM.pdf]

Appendix 3 - Physician Organization Qualitative Interview Guide

Physician Organization Name ______________________________

Date of Call ______________________________

Individuals on the call: ______________________________

1. Domain: Inner Setting.

Tell me a little bit about your physician organization (e.g, values, size, leadership, structure). ______________________________

2. Domain: Outer Setting.

What is unique about the community that your PO and practices serve? What aspects of your community would be particularly important as we roll out the MCT2D program? (.e.g, high rates of diabetes, area of high poverty) ______________________________

3. Domain: Inner Setting.

How often do you communicate with your practices (e.g., quarterly, monthly)? How do you generally communicate with your practices (e.g, emails, remote meetings)? What kinds of information do you tend to share with your practices (e.g., performance data)? ______________________________

4. Domain: Intervention Characteristics.

How compatible is MCT2D participation with the priorities and ongoing operations of your PO? ______________________________

a) Do you have any other current projects in your PO related to diabetes care? If so, what are they? ______________________________

b) How do the interventions that MCT2D is planning to implement compare to other strategies that you may have? ______________________________

5. Domain: Intervention Characteristics.

What do you anticipate being the largest challenge for your PO related to MCT2D participation? ______________________________

a) What do you perceive as the biggest challenges related to high quality type 2 diabetes care? ______________________________

b) Does MCT2D address these challenges to high quality diabetes care? If so, how?

---

6. Domain: Intervention Characteristics.

Considering the MCT2D approach to improving type 2 diabetes care through increasing prescribing of SGLT2s/GLP1s and using continuous glucose monitoring devices with lower-carbohydrate diets, what other approaches do you think we need to implement to be successful in improving T2D care?

---

7. Domain: Process.

What diabetes-related personnel do you currently have at your PO (e.g. diabetes educators, dieticians, clinical pharmacists, etc.)?

---

a) Tell me more about how these resources support T2D Care.

---

b) Do you plan to bring on additional resources with the funding provided by BCBSM?

---

8. Domain: Process.

Are you currently submitting the All-Payer Supplemental (APS) clinical data to MiHIN as part of the BCBS PPQC (Payer-Provider Quality Collaborative)?

---

a) Is this data being submitted for all practices eligible for participation in MCT2D?

If not, approximately how many?

---

b) Do you limit the data in any way? (i.e. only some payers)

---

c) If you are not currently submitting data, do you plan to start to submit it?

If so, do you have a timeline?

---

d) If not, what are some barriers to submission?

---

9. Domain: Process.

Does your organization currently send CCDAs to MiHIN?

a) If yes, are CCDAs submitted for all practices eligible for participation in MCT2D?

b) If you are not currently submitting data, or a portion of practices are not, do you have plans to submit it going forward? If yes, what is your timeline?

c) If no, what are the barriers to submission of this data?

10. Domain: Outer Setting.

Like other collaborative quality initiative programs, MCT2D has a coordinating center that will provide guidance on the implementation of quality improvement initiatives, work with your practices to address
barriers, and report on performance. Given this description of the coordinating center, how can the coordinating center best support your PO and your practices to successfully implement the MCT2D program?

11. Domain: Outer Setting.

As you may recall in the description of the PO expectations, Blue Cross is asking each participating PO to partner with a hospital. How could your hospital partner best support the work that your PO will be doing as part of MCT2D?

12. Domain: Characteristics of Individuals.

Could you rate on a scale of 1-10 how confident you are that your PO and practices will be able to successfully implement the MCT2D interventions?

a) What made you choose that number?
