# Supplementary material for: The Michigan Collaborative for Type 2 Diabetes (MCT2D): Development and implementation of a statewide collaborative quality initiative
Source: BMC Health Serv Res. 2024 Oct 17;24:1254. doi: 10.1186/s12913-024-11520-z (PMC11487891; doi:10.1186/s12913-024-11520-z)
Supplement: Supplementary file 4 — Supplementary Material 4: Appendix 4 [file 12913_2024_11520_MOESM4_ESM.pdf]

Appendix 4: Practice Change Readiness Assessment Quantitative Survey Questions

Are you aware of what the MCT2D Collaborative Quality Initiative is?

- Very Aware
- Mostly Aware
- Moderately Aware
- Somewhat Aware
- Not At All Aware

Has the Practice Clinical Champion reviewed the practice Introduction to MCT2D PowerPoint and familiarized themselves with the approach and quality improvement initiatives?

- Yes
- No

Do you understand the objectives and goals of the MCT2D project?

- Very Well
- Mostly
- Moderately
- Somewhat
- Not At All

How confident are you that your practice will be able to implement the Continuous Glucose Monitoring quality improvement initiative?

- Very Confident
- Mostly Confident
- Moderately Confident
- Somewhat Confident
- Not At All Confident

How confident are you that your practice will be able to implement the medication (increased use of GLP1-RA and SGLT2-I) quality improvement initiative?

- Very Confident
- Mostly Confident
- Moderately Confident
- Somewhat Confident
- Not At All Confident

How confident are you that your practice will be able to implement the low carbohydrate eating pattern quality improvement initiative?

- Very Confident
- Mostly Confident
- Moderately Confident
- Somewhat Confident
- Not At All Confident

In your opinion, how committed is your physician organization to the success of the MCT2D project?

- Very Confident
- Mostly Confident
- Moderately Confident
- Somewhat Confident
- Not At All Confident

How effective is communication from the Physician Organization to your practice?

- Very Effective
- Mostly Effective
- Moderately Effective
- Somewhat Effective
- Not At All Effective

Do you know what person at the Physician Organization you would contact if you had questions about MCT2D?

- Yes
- No

In your opinion, do people at your practice know whom to ask when they need help improving quality?

- Yes
- No

How confident are you that your Physician Organization will provide the support you need to fulfill the goals of MCT2D?

- Very Confident
- Mostly Confident
- Moderately Confident
- Somewhat Confident
- Not At All Confident

Does your clinic employ a Clinical Pharmacist or routinely refer patients with Type 2 Diabetes to a Clinical Pharmacist?

- Yes, we employ a clinical pharmacist
- Yes, we routinely refer to a clinical pharmacist outside of our practice

- No

Does your clinic currently refer patients to anyone for nutrition counseling?

- Yes, we employ a nutritionist/dietician
- Yes, we routinely refer to a nutritionist/dietician outside of our practice
- No

What is the name of the person you foresee your patients receiving their low carb diet education from?
Has anyone in your practice ever prescribed a continuous glucose monitor (CGM) for a patient with type 2 diabetes?

- Yes
- No

Has your practice ever received CGM data from a patient before?

- Yes
- No

Does your practice have a process for getting CGM data from patients?

- Yes
- No

What is your workflow for getting CGM data from patients?

What do you find to be the largest barrier(s) in prescribing SGLT2-Is/GLP1-RAs to your patients? (Check all that apply)

- - Cost of medication to patient
- - Concern about side effects of adverse events
- - Coverage issues and challenges (e.g. prior authorizations_
- - Injectable hesitance
- - Other _______________

If Other, please describe.
